# The wiener index of the zero-divisor graph for a new class of residue class rings

**DOI:** 10.3389/fchem.2022.985001

**Published:** 2022-09-13

**Authors:** Yinhu Wei, Ricai Luo

**Affiliations:** School of Mathematics and Physics, Hechi University, Yizhou, China

**Keywords:** wiener index, zero-divisor graphs, compressed zero-divisor graph, residue class rings, equivalence classification

## Abstract

The zero-divisor graph of a commutative ring R, denoted by Γ(*R*), is a graph whose two distinct vertices *x* and *y* are joined by an edge if and only if *xy* = 0 or *yx* = 0. The main problem of the study of graphs defined on algebraic structure is to recognize finite rings through the properties of various graphs defined on it. The main objective of this article is to study the Wiener index of zero-divisor graph and compressed zero-divisor graph of the ring of integer modulo *p* ^*s*^ *q* ^*t*^ for all distinct primes *p*, *q* and s,t∈N . We study the structure of these graphs by dividing the vertex set. Furthermore, a formula for the Wiener index of zero-divisor graph of Γ(*R*), and a formula for the Wiener index of associated compressed zero-divisor graph Γ_*E*_(*R*) are derived for $R=Z_{p^{s}q^{t}}$ .

## Introduction

The study of graphs defined on algebraic structures has been an active topic of research in the last few decades. The main question in the area is to recognize finite rings through the properties of various graphs defined on it. The notion of the zero-divisor graph of a commutative ring was introduced by I. Beck in (Beck, 1988), where he considered the set of zero divisors including zero and introduced the concepts such as diameter, grith and clique number of a zero divisor graph. Then later on in (Anderson and Livingston, 1999), Anderson and Livingston changed the vertex set of the zero-divisor graph, they considered only the vertices of the non-zero zero-divisors. For more details, one may see the survey (Singh and Bhat, 2020) and the references therein for the vast literature on the study of zero-divisor graphs.

The Wiener index is one of the important graph indices, and has a variety of applications in pharmaceutical science and in the structure of nanotubes. For results and applications of Wiener index, see (Devillers and Balaban, 1999; Dobrynin et al., 2001; Dehmer and Emmert-Streib, 2014; Dobrynin and Iranmanesh, 2020). There are some works of the Wiener index were done for the ring of integers modulo *n*. Let us review some of the work done on the topological indices of the zero-divisor graphs. Let *p*, *q* be distinct prime numbers. Ahmadi et al. (Ahmadi and Nezhad, 2011) in 2011 has provided an algorithm to determining the Wiener index of $Z_{n}$ for *n* = *p* ^2^, *pq*. In 2018, Mohammad et al. (Mohammad and Authman, 2018) has extended the result by determining the Wiener index of a zero-divisor graph of $\Gamma \left(Z_{n}\right)$ for *n* = *p* ^*m*^ and *p* ^*m*^ *q*, where m∈Z and *m* ≥ 2 using the Hosoya polynomial. Pirzada et al. (Pirzada et al., 2020) in 2020 determined the Wiener index of a zero-divisor graph and a compressed zero-divisor of $Z_{p^{m}}$ for m∈N . In (Asir and Rabikka, 2021), recently a constructed method to calculate the Wiener index of zero-divisor graph of $Z_{n}$ for any positive integer *n* is determined. The authors of (Asir and Rabikka, 2021) calculated the complete formula through restrict *n* as product of distinct primes and the remaining cases. In 2022, Selvakumar et al. (Selvakumar et al., 2022) visualized the zero-divisor graph Γ(*R*) as a generalized composition of suitable choices of graphs and derived a formula for the Wiener index of the graph $\Gamma \left(Z_{n}\right)$ .

In this paper, we are interested in the parameter Wiener index of graphs for the rings of integers modulo *p* ^*s*^ *q* ^*t*^. Although the formulas in the general case for the rings of $Z_{n}$ have been obtained in literatures (Asir and Rabikka, 2021) and (Selvakumar et al., 2022), compared with their results, our formula is more direct and convenient for calculation the Wiener index $W\left(\Gamma \left(Z_{p^{s}q^{t}}\right)\right)$ . We also get the formula for compressed zero-divisor graph.

## Preliminaries

Throughout this paper we assume that *R* denotes a commutative ring with identity, *Z*(*R*) be its set of zero-divisors, the (nonempty) set of nonzero zero-divisors and unit elements denoted by *Z*(*R*)* and *U*(*R*). We use Z to note the ring of integers.

Definition 1 *Let* *G* *be a graph and let* *u* *and* *v* *be two vertices of* *G* *. The distance between* *u* *and* *v* *, denoted by* *d*_*G*_(*u*, *v*)*, is defined to be the length of the shortest path between* *u* *and* *v* *. The Wiener index of the graph* *G* *, denoted by* *W*(*G*)*, is defined to be the sum of all distanced between any two vertices of* *G* *.*

Let *d*_*G*_(*v*) denote the sum of distances of the vertex *v* from all the vertices of *G*, then the Wiener index can be redefined as

$$W\left(G\right)=\frac{1}{2}\underset{v\in V\left(G\right)}{\sum }d_{G}\left(v\right).$$

Let *R* be an arbitrary finite commutative ring with unity. We define an equivalence relation ∼ on *Z*(*R*)* as follows. For *x*, *y* ∈ *Z*(*R*)*, define *x* ∼ *y* if and only if *ann*(*x*) = *ann*(*y*) where *ann*(*x*) = {*r* ∈ *R*|*rx* = 0}. We call these classes the equiv-annihilator classes of the zero-divisor graph Γ(*R*).

We write *d* (*x*, *y*) to denote the distance between *x* and *y* in *Z*(*R*)*, and write *x* ∼ *y* to denote *x* and *y* are adjacent, otherwise *x* ≁ *y*. Let *U*, *V* be subsets of the vertex of Γ(*R*), the *U* ↔ *V* shall denote that each vertex of *U* is adjacent to every vertex of *V*, and *U* ↮ *V* denotes that no vertex of *U* is adjacent to every vertex of *V*.

The so-called compressed zero-divisor graph of a ring was first defined by the Spiroff et al. in (Spiroff and Wickham, 2011).

Definition 2 *For a commutative ring* *R* *with* 1 ≠ 0*, a compressed zero-divisor graph of a ring* *R* *is the undirected graph* Γ_*E*_(*R*) *with vertex set* *Z*(*R*_*E*_) − [0] = *R*_*E*_ − {[0], [1]} *defined by* *R*_*E*_ = {[*x*]|*x* ∈ *R*}*, where* [*x*] = {*y* ∈ *R*|*ann*(*x*) = *ann*(*y*)} *and two distinct vertices* [*x*] *and [y] are adjacent if and only if* [*x*][*y*] = [0] = [*xy*]*, that is, if and only if* *xy* = 0*.*

In what follows, we use the graph-theoretic notions from (Douglas, 2001).

## Main results

In this section, we first give a structure of $R=Z_{p^{s}q^{t}}$ using the method of equivalence classification.

Let *p*, *q* be distinct prime numbers and s,t∈N , the vertex set of $R=Z_{p^{s}q^{t}}$ be divided into disjoint subsets *V*_00_, …, *V*_*ij*_, …, *V*_*st*_, where

$$V_{ij}=\left\{\begin{array}{cc} \left\{kp^{i}q^{j}\in Z_{n}|p∤k\;and\;q∤k\right\}\; & if\;i<s\;and\;j<t\\ \left\{kp^{i}q^{t}\in Z_{n}|p∤k\right\}\;\; & if\;i<s\;and\;j=t\\ \left\{kp^{s}q^{j}\in Z_{n}|q∤k\right\}\;\; & if\;i=s\;and\;j<t. \end{array}\right.$$ (1)

We noted that *V*_*st*_ =∅ and $V_{00}⊈Z{\left(Z_{p^{s}q^{t}}\right)}^{*}$ . For the convenience of presentation, we always assumes that *V*_00_ and *V*_*st*_ are empty sets in the following, unless otherwise specified. Therefore

$$V\left(\Gamma \left(Z_{p^{s}q^{t}}\right)\right)=\underset{\begin{array}{c} 0\leq i\leq s \end{array}}{⋃}\left(\underset{0\leq j\leq t}{⋃}V_{ij}\right).$$

Example 1 *Consider the ring* $R=Z_{2^{2}\times 3^{2}}$ *. The vertex set of* $\Gamma \left(Z_{2^{2}\times 3^{2}}\right)$ *is*

$$\begin{array}{ll} V\left(\Gamma \left(Z_{2^{2}\times 3^{2}}\right)\right) & =V_{01}⋃V_{02}⋃V_{10}⋃V_{11}⋃V_{12}⋃V_{20}⋃V_{21}\\ & =\left\{3,15,21,33\right\}⋃\left\{9,27\right\}⋃\left\{2,10,14,22,26,34\right\}\\ & \;⋃\left\{6,30\right\}⋃\left\{18\right\}⋃\left\{4,8,16,20,28,32\right\}⋃\left\{12,24\right\}. \end{array}$$

It is not difficult to see that *V*_*ij*_ be the equiv-annihilator classes of $\Gamma \left(Z_{p^{s}q^{t}}\right)$ , where 0 ≤ *i* ≤ *s* and 0 ≤ *j* ≤ *t*. If *i* < *s* and *j* < *t*, for any *x*, *y* ∈ *V*_*ij*_. Let *z* ∈ *ann*(*x*), then *z* = *k*′*p* ^*s*−*i*^ *q* ^*t*−*j*^. So *yz* = (*kp* ^*i*^ *q* ^*j*^) (*k*′*p* ^*s*−*i*^ *q* ^*t*−*j*^) = *kk*′*p* ^*s*^ *q* ^*t*^, that is, *z* ∈ *ann*(*y*). If *i* < *s* and *j* = *t*, for any *x*, *y* ∈ *V*_*ij*_. Let *z* ∈ *ann*(*x*), then *z* = *k*′*p* ^*s*−*i*^. So *yz* = (*kp* ^*i*^ *q* ^*t*^) (*k*′*p* ^*s*−*i*^) = *kk*′*p* ^*s*^ *q* ^*t*^, that is, *z* ∈ *ann*(*y*). If *i* = *s* and *j* < *t*, for any *x*, *y* ∈ *V*_*ij*_. Let *z* ∈ *ann*(*x*), then *z* = *k*′*q* ^*t*−*j*^. So *yz* = (*kp* ^*s*^ *q* ^*j*^) (*k*′*q* ^*t*−*j*^) = *kk*′*p* ^*s*^ *q* ^*t*^, that is, *z* ∈ *ann*(*y*). Thus *ann*(*x*) = *ann*(*y*) for any *x*, *y* ∈ *V*_*ij*_.

Next, we prove some elementary properties of the vertex subsets *V*_*ij*_.

Lemma 1 *For distinct prime numbers* *p*, *q* *, let* *n* = *p* ^*s*^ *q* ^*t*^ *for some* s,t∈N *and* *V*_*ij*_ *be the equiv-annihilator classes of* $\Gamma \left(Z_{n}\right)$ *where* 0 ≤ *i* ≤ *s* *and* 0 ≤ *j* ≤ *t* *. Then*

(1) $|V_{ij}|=\left\{\begin{array}{cc} \left(p-1\right)p^{s-i-1}\left(q-1\right)q^{t-j-1}\;\; & if\;i\neq s\;and\;j\neq t\\ \left(q-1\right)q^{t-j-1}\;\; & if\;i=s\\ \left(p-1\right)p^{s-i-1}\;\; & if\;j=t. \end{array}\right.$

(2) *V*_*ij*_ ↔ *V*_*i*′*j*′_ *if and only if* *i* + *i*′ ≥ *s* *and* *j* + *j*′ ≥ *t* *.*

Proof (1) we consider the following cases.

**Case 1:** *i* ≠ *s* and *j* ≠ *t*.

Let *S*_*ij*_ be the set of all the elements that can be divisible by *p* ^*i*^ *q* ^*j*^ in $Z_{n}$ . By the inclusion-exclusion principle,

$$|V_{ij}|=|S_{ij}|-|pS_{ij}|-|qS_{ij}|+|pqS_{ij}|.$$

Note that |*S*_*ij*_| = |{*kp* ^*i*^ *q* ^*j*^|0 ≤ *k* < *p* ^*s*−*i*^ *q* ^*t*−*j*^}| = *p* ^*s*−*i*^ *q* ^*t*−*j*^. Since

$$|pS_{ij}|=|\left\{kp^{i+1}q^{j}|0\leq k<p^{s-i-1}q^{t-j}\right\}|=p^{s-i-1}q^{t-j}$$

$$|qS_{ij}|=|\left\{kp^{i}q^{j+1}|0\leq k<p^{s-i}q^{t-j-1}\right\}|=p^{s-i}q^{t-j-1}$$

And

$$|pqS_{sj}|=|\left\{kp^{i+1}q^{j+1}|0\leq k<p^{s-i-1}q^{t-j-1}\right\}|=p^{s-i-1}q^{t-j-1}.$$

Then

$$\begin{array}{ll} \left|V_{ij}\right| & =p^{s-i}q^{t-j}-p^{s-i-1}q^{t-j}-p^{s-i}q^{t-j-1}+p^{s-i-1}q^{t-j-1}\\ & =\left(p-1\right)p^{s-i-1}\left(q-1\right)q^{t-j-1}. \end{array}$$

**Case 2:** *i* = *s*.

Since

$$\left|S_{sj}|=|\left\{kp^{s}q^{j}|0\leq k<q^{t-j}\;and\;q∤k\right\}\right|$$

Then

$$|S_{sj}|=q^{t-j}-q^{t-j-1}=\left(q-1\right)q^{t-j-1}.$$

**Case 3:** *j* = *t*.

Since

$$\left|S_{it}|=|\left\{kp^{i}q^{t}|0\leq k<p^{s-i}\;and\;p∤k\right\}\right|$$

Then

$$|S_{it}|=p^{s-i}-p^{s-i-1}=\left(p-1\right)p^{s-i-1}.$$

(2) Let *x* = *k*_*ij*_ *p* ^*i*^ *q* ^*j*^ ∈ *V*_*ij*_, *y* = *k*_*i*′*j*′_ *p* ^*i*^′*q* ^*j*^′ ∈ *V*_*i*′*j*′_. If *i* + *i*′ ≥ *s* and *j* + *j*′ ≥ *t*, then

$$xy=k_{ij}k_{i^{\prime }j^{\prime }}p^{i+i^{\prime }}q^{j+j^{\prime }}=k_{ij}k_{i^{\prime }j^{\prime }}p^{i+i^{\prime }-s}q^{j+j^{\prime }-t}n\equiv 0\left(modn\right).$$

So *x* is adjacent to *y*.

Conversely, suppose *V*_*ij*_ ↔ *V*_*i*′*j*′_. If *i* + *i*′ < *s* or *j* + *j*′ < *t*. We have *xy* = *k*_*ij*_ *k*_*i*′*j*′_ *p* ^*i*+*i*^′*q* ^*j*+*j*^′ can’t be a multiple of *n*, a contradiction.

The following result characterized the distance between the equiv-annihilator classes.

Proposition 1 *For distinct prime numbers* *p*, *q* *, let* $x,y\in V\left(\Gamma \left(Z_{p^{s}q^{t}}\right)\right)$ *for some* s,t∈N *. Then* *d*(*x*, *y*) = 1, 2 *or* 3*.*

Proof. Let *V*_01_, *V*_10_, ⋯ , *V*_*s*,*t*−1_, *V*_*s*−1,*t*_ be the equiv-annihilator classes of $\Gamma \left(Z_{p^{s}q^{t}}\right)$ , where *V*_*ij*_ defined by (1). For $x\in V_{i_{1}j_{1}}$ and $y\in V_{i_{2}j_{2}}$ , where 0 ≤ *i*_1_, *i*_2_ ≤ *s* and 0 ≤ *j*_1_, *j*_2_ ≤ *t*.

If *i*_1_ + *i*_2_ ≥ *s* and *j*_1_ + *j*_2_ ≥ *s*, then *x* ∼ *y* and *d* (*x*, *y*) = 1 by lemma 1. So we only need to consider the cases of *i*_1_ + *i*_2_ < *s* or *j*_1_ + *j*_2_ < *s* in the following, that is, *x* ≁ *y*. Without loss of generality,we may assume that *i*_1_ + *i*_2_ < *s*. Consider the following cases.

**Case 1:** 0 < *i*_1_, *i*_2_ < *s*.

Let *i* = *s* − *min*{*i*_1_, *i*_2_}, *j* = *t*. We have *i*_1_ + *i* ≥ *s* and *j*_1_ + *j* ≥ *t*, also *i* + *i*_2_ ≥ *s* and *j* + *j*_2_ ≥ *t*. Then $V_{i_{1}j_{1}}↔V_{ij}↔V_{i_{2}j_{2}}$ . Hence, *d* (*x*, *y*) = 2.

**Case 2:** *i*_1_ = 0 and *i*_2_ = 0.

Let *i* = *s*, *j* = *t* − *min*{*j*_1_, *j*_2_}. We have *i*_1_ + *i* ≥ *s* and *j*_1_ + *j* ≥ *t*, also *i* + *i*_2_ ≥ *s* and *j* + *j*_2_ ≥ *t*. Then $V_{i_{1}j_{1}}↔V_{ij}↔V_{i_{2}j_{2}}$ . Hence, *d* (*x*, *y*) = 2.

**Case 3:** *i*_1_ = 0 and *i*_2_ ≠ 0. Consider the following subcases.

Subcase3.1: If *j*_2_ = 0. Let *i*_3_ = *s*, *i*_4_ = *s* − *i*_2_, *j*_3_ = *t* − *j*_1_, and *j*_4_ = *t*. We have

$$i_{1}+i_{3}=s,\;i_{2}+i_{4}=s,\;i_{3}+i_{4}=s+\left(s-i_{2}\right)>s$$

And

$$j_{1}+j_{3}=t,\;j_{2}+j_{4}=t,\;j_{3}+j_{4}=\left(t-j_{1}\right)+t>t.$$

Thus $V_{i_{1}j_{1}}↔V_{i_{3}j_{3}}↔V_{i_{4}j_{4}}↔V_{i_{2}j_{2}}$ .

Since

$$i_{1}+i_{4}=0+\left(s-i_{2}\right)<s,\;j_{3}+j_{2}=\left(t-j_{1}\right)+0<t,$$

Then $V_{i_{1}j_{1}}↮V_{i_{4}j_{4}}$ and $V_{i_{3}j_{3}}↮V_{i_{2}j_{2}}$ . Therefore, *d* (*x*, *y*) = 3.

Subcase3.2: If *j*_2_ ≠ 0. Let *i* = *s* and *j* = *t* − *min*{*j*_1_, *j*_2_}. We have

$$i_{1}+i=s,\;j_{1}+j\geq t$$

And

$$i+i_{2}>s,\;j+j_{2}\geq t.$$

Thus $V_{i_{1}j_{1}}↔V_{ij}↔V_{i_{2}j_{2}}$ . Therefore, *d* (*x*, *y*) = 2.

**Case 4:** *i*_1_ ≠ 0 and *i*_2_ = 0. A similar argument as in Case 3 shows that *d* (*x*, *y*) = 2 *or* 3.

We have already shown that in any case, *d* (*x*, *y*) = 1, 2 *or* 3.

Now, we can calculate the Wiener index of $\Gamma \left(Z_{p^{s}q^{t}}\right)$ .

Theorem 1 *For distinct prime numbers* *p*, *q* *, and some* s,t∈N *. The Wiener index*

$$\begin{array}{cc} W\left(\Gamma \left(Z_{p^{s}q^{t}}\right)\right) & =\overset{\left\lceil \frac{s}{2}\right\rceil -1}{\underset{i=0}{\sum }}\overset{t}{\underset{j=0}{\sum }}|V_{ij}|\left(|V_{ij}|-1\right)+\overset{s}{\underset{i=0}{\sum }}\overset{\left\lceil \frac{t}{2}\right\rceil -1}{\underset{j=0}{\sum }}|V_{ij}|\left(|V_{ij}|-1\right)-\overset{\left\lceil \frac{s}{2}\right\rceil -1}{\underset{i=0}{\sum }}\overset{\left\lceil \frac{t}{2}\right\rceil -1}{\underset{j=0}{\sum }}|V_{ij}\\ & \;\left(|V_{ij}|-1\right)+\overset{s}{\underset{i=\left\lceil \frac{s}{2}\right\rceil }{\sum }}\overset{t}{\underset{j=\left\lceil \frac{t}{2}\right\rceil }{\sum }}\frac{|V_{ij}|\left(|V_{ij}|-1\right)}{2}+2\overset{s}{\underset{i=0}{\sum }}\overset{t}{\underset{j=0}{\sum }}|V_{ij}|\\ & \;\left(\overset{t}{\underset{j^{\prime }=j+1}{\sum }}|V_{ij^{\prime }}|+\overset{s}{\underset{i^{\prime }=i+1}{\sum }}\overset{t}{\underset{j^{\prime }=0}{\sum }}|V_{i^{\prime }j^{\prime }}|\right)-\overset{s}{\underset{i=\left\lceil \frac{s}{2}\right\rceil }{\sum }}\overset{t}{\underset{j=0}{\sum }}|V_{ij}|\\ & \;\left(\overset{t}{\underset{j^{\prime }=\max\left\{t-j,j+1\right\}}{\sum }}|V_{ij^{\prime }}|\right)-\overset{s-1}{\underset{i=0}{\sum }}\overset{t}{\underset{j=0}{\sum }}|V_{ij}|\left(\overset{s}{\underset{i^{\prime }=\max\left\{s-i,i+1\right\}}{\sum }}\overset{t}{\underset{j^{\prime }=t-j}{\sum }}|V_{i^{\prime }j^{\prime }}|\right)\\ & \;+\overset{t}{\underset{j=0}{\sum }}\overset{s}{\underset{i^{\prime }=0}{\sum }}|V_{0j}\|V_{i^{\prime }0}|-|V_{s0}\|V_{0t}| \end{array}$$

where

$|V_{ij}|=\left\{\begin{array}{cc} \left(p-1\right)p^{s-i-1}\left(q-1\right)q^{t-j-1}\;\; & if\;i\neq s\;and\;j\neq t\\ \left(q-1\right)q^{t-j-1}\;\; & if\;i=s\\ \left(p-1\right)p^{s-i-1}\;\; & if\;j=t. \end{array}\right.$

Proof. Let *n* = *p* ^*s*^ *q* ^*t*^, we have *V*_01_, *V*_10_, …, *V*_*s*−1,*t*_, *V*_*s*,*t*−1_ is the partition of $V\left(\Gamma \left(Z_{p^{s}q^{t}}\right)\right)$ ,where *V*_*ij*_ defined by (1). For any two different elements *x*, *y* in *V*_*ij*_. By the proof of Proposition 1, there are the following cases.

**Case 1:** $0\leq i\leq \left\lceil \frac{s}{2}\right\rceil -1$ or $0\leq j\leq \left\lceil \frac{t}{2}\right\rceil -1$ .

In this case, we have *d* (*x*, *y*) = 2. Then

$$\begin{array}{ll} \underset{x,y\in V_{ij}}{\sum }d\left(x,y\right) & =\overset{\left|V_{ij}\right|}{\underset{k=2}{\sum }}d\left(x_{1},x_{k}\right)+\overset{\left|V_{ij}\right|}{\underset{k=3}{\sum }}d\left(x_{2},x_{k}\right)+\cdots +d\left(x_{|V_{ij}|-1},x_{\left|V_{ij}\right|}\right)\\ & =2\left(|V_{ij}|-1\right)+2\left(|V_{ij}|-2\right)+\cdots +2\\ & =|V_{ij}|\left(|V_{ij}|-1\right). \end{array}$$

**Case 2:** $\left\lceil \frac{s}{2}\right\rceil \leq i\leq s$ and $\left\lceil \frac{t}{2}\right\rceil \leq j\leq t$ .

In this case, *d* (*x*, *y*) = 1. Then

$$\begin{array}{ll} \underset{x,y\in V_{ij}}{\sum }d\left(x,y\right) & =\overset{\left|V_{ij}\right|}{\underset{k=2}{\sum }}d\left(x_{1},x_{k}\right)+\overset{\left|V_{ij}\right|}{\underset{k=3}{\sum }}d\left(x_{2},x_{k}\right)+\cdots +d\left(x_{|V_{ij}|-1},x_{\left|V_{ij}\right|}\right)\\ & =\left(|V_{ij}|-1\right)+\left(|V_{ij}|-2\right)+\cdots +1\\ & =\frac{|V_{ij}|\left(|V_{ij}|-1\right)}{2}. \end{array}$$

Let *x* and *y* be the elements in the two different equiv-annihilator classes, *V*_*ij*_ and *V*_*i*′*j*′_, respectively. Consider the following cases.

**Case 3:** *i* + *i*′ ≥ *s* and *j* + *j*′ ≥ *t*.

By Lemma 1, *d* (*x*, *y*) = 1. Then

$$\underset{x\in V_{ij}}{\sum }\underset{y\in V_{i^{\prime }j^{\prime }}}{\sum }d\left(x,y\right)=|V_{ij}\|V_{i^{\prime }j^{\prime }}|.$$

**Case 4:** 0 < *i* + *i*′ < *s* or 0 < *j* + *j*′ < *t*.

Subcase **4.1:** *i* = 0 and *j*′ = 0.

In this case, we have *d* (*x*, *y*) = 3. Hence

$$\underset{x\in V_{ij}}{\sum }\underset{y\in V_{i^{\prime }j^{\prime }}}{\sum }d\left(x,y\right)=3|V_{ij}\|V_{i^{\prime }j^{\prime }}|.$$

**Subcase 4.2:** *i*′ = 0 and *j* = 0.

In this case, *d* (*x*, *y*) = 3. Hence

$$\underset{x\in V_{ij}}{\sum }\underset{y\in V_{i^{\prime }j^{\prime }}}{\sum }d\left(x,y\right)=3|V_{ij}\|V_{i^{\prime }j^{\prime }}|.$$

**Subcase 4.3:** If *i*, *j*′ are not both equal to 0, and *i*′, *j* are not both equal to 0.

In this case, *d* (*x*, *y*) = 2. Hence

$$\underset{x\in V_{ij}}{\sum }\underset{y\in V_{i^{\prime }j^{\prime }}}{\sum }d\left(x,y\right)=2|V_{ij}\|V_{i^{\prime }j^{\prime }}|.$$

In conclusion, the Weiner index is

$$\begin{array}{ll} W\left(\Gamma \left(Z_{p^{s}q^{t}}\right)\right. & =\overset{s}{\underset{i=0}{\sum }}\overset{t}{\underset{j=0}{\sum }}\left(\underset{x,y\in V_{ij}}{\sum }d\left(x,y\right)\right)+\overset{s}{\underset{i,i^{\prime }=0}{\sum }}\overset{t}{\underset{j,j^{\prime }=0}{\sum }}\left(\underset{x\in V_{ij}}{\sum }\underset{y\in V_{i^{\prime }j^{\prime }}}{\sum }d\left(x,y\right)\right)\\ & =\overset{\left\lceil \frac{s}{2}\right\rceil -1}{\underset{i=0}{\sum }}\overset{t}{\underset{j=0}{\sum }}|V_{ij}|\left(|V_{ij}|-1\right)+\overset{s}{\underset{i=0}{\sum }}\overset{\left\lceil \frac{t}{2}\right\rceil -1}{\underset{j=0}{\sum }}|V_{ij}|\left(|V_{ij}|-1\right)-\overset{\left\lceil \frac{s}{2}\right\rceil -1}{\underset{i=0}{\sum }}\overset{\left\lceil \frac{t}{2}\right\rceil -1}{\underset{j=0}{\sum }}|V_{ij}|\\ & \;\left(|V_{ij}|-1\right)+\overset{s}{\underset{i=\left\lceil \frac{s}{2}\right\rceil }{\sum }}\overset{t}{\underset{j=\left\lceil \frac{t}{2}\right\rceil }{\sum }}\frac{|V_{ij}|\left(|V_{ij}|-1\right)}{2}+2\overset{s}{\underset{i=0}{\sum }}\overset{t}{\underset{j=0}{\sum }}|V_{ij}|\\ & \;\left(\overset{t}{\underset{j^{\prime }=j+1}{\sum }}|V_{ij^{\prime }}|+\overset{s}{\underset{i^{\prime }=i+1}{\sum }}\overset{t}{\underset{j^{\prime }=0}{\sum }}|V_{i^{\prime }j^{\prime }}|\right)-\overset{s}{\underset{i=\left\lceil \frac{s}{2}\right\rceil }{\sum }}\overset{t}{\underset{j=0}{\sum }}|V_{ij}|\\ & \;\times \left(\overset{t}{\underset{j^{\prime }=\max\left\{t-j,j+1\right\}}{\sum }}|V_{ij^{\prime }}|\right)-\overset{s}{\underset{i=0}{\sum }}\overset{t}{\underset{j=0}{\sum }}|V_{ij}|\left(\overset{s}{\underset{i^{\prime }=\max\left\{s-i,i+1\right\}}{\sum }}\overset{t}{\underset{j^{\prime }=t-j}{\sum }}|V_{i^{\prime }j^{\prime }}|\right)\\ & \;+\overset{t}{\underset{j=0}{\sum }}\overset{s}{\underset{i^{\prime }=0}{\sum }}|V_{0j}\|V_{i^{\prime }0}|-|V_{s0}\|V_{0t}|. \end{array}$$

Therefore the result holds, by Lemma 1.

The following Table gives the exact value of $W\left(\Gamma \left(Z_{n}\right)\right)$ for *n* = 2^*s*^3^*t*^, where 1 ≤ *s* ≤ 3 and 1 ≤ *t* ≤ 3.

The compressed zero-divisor graph of $Z_{p^{s}q^{t}}$ can be obtained by treating the set *V*_*ij*_, 0 ≤ *i* ≤ *s*, 0 ≤ *j* ≤ *t*, as a single vertex. To illustrate, let’s give an example in the following.

**FIGURE 1:**
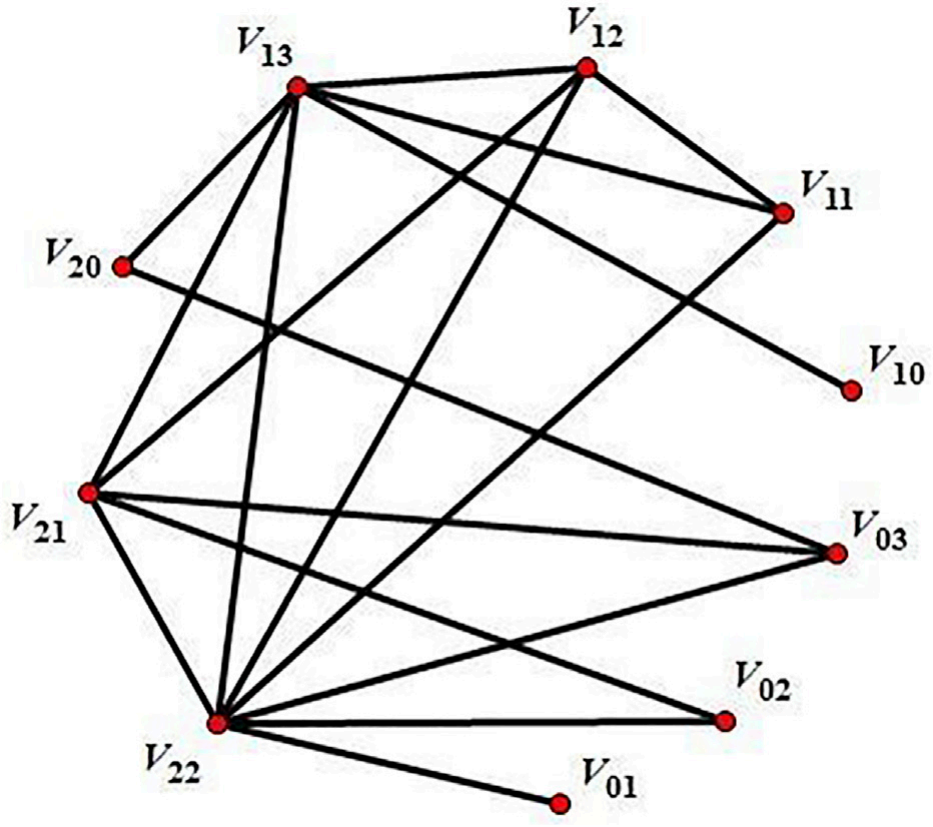
the compressed zero-divisor graph $\Gamma_{E}\left(Z_{2^{2}\times 3^{3}}\right)$ .

Example 2 *Consider the ring* $R=Z_{2^{2}\times 3^{3}}$ *, the vertex set of* $\Gamma \left(Z_{2^{2}\times 3^{3}}\right)$ *is divided into 10 sets* *V*_01_ *,* *V*_02_ *,* *V*_03_ *,* *V*_10_ *,* *V*_11_ *,* *V*_12_ *,* *V*_13_ *,* *V*_20_ *,* *V*_21_ *,* *V*_22_ *. Then the associated compressed zero-divisor graph* $\Gamma_{E}\left(Z_{2^{2}\times 3^{3}}\right)$ *is shown in* Figure 1.

Before proving the next result we need the following lemma.

Lemma 2 *For distinct prime numbers* *p*, *q* *, let* *n* = *p* ^*s*^ *q* ^*t*^ *for some* s,t∈N *and* $G=\Gamma_{E}\left(Z_{n}\right)$ *be the compressed zero-divisor graph of* $Z_{n}$ *. Then*

(1) *V*(*G*) = {*V*_*ij*_|0 ≤ *i* ≤ *s*, 0 ≤ *j* ≤ *t*} *.*
(2) $d_{G}\left(V_{ij}\right)=\left\{\begin{array}{cc} 2\left(s+1\right)\left(t+1\right)+s-j-6\;\; & if\;i=0\;and\;0<j<t\\ 2\left(s+1\right)\left(t+1\right)+s-t-7\;\; & if\;i=0\;and\;j=t\\ 2\left(s+1\right)\left(t+1\right)+t-i-6\;\; & if\;0<i<t\;and\;j=0\\ 2\left(s+1\right)\left(t+1\right)+t-s-7\;\; & if\;i=s\;and\;j=0\\ 2\left(s+1\right)\left(t+1\right)-\left(i+1\right)\left(j+1\right)-4\;\; & if\;i\geq \left\lceil \frac{s}{2}\right\rceil \;and\;j\geq \left\lceil \frac{t}{2}\right\rceil \\ 2\left(s+1\right)\left(t+1\right)-\left(i+1\right)\left(j+1\right)-5\;\; & otherwise. \end{array}\right.$

Proof (1) Note that

$$Z{\left(Z_{n}\right)}^{*}=\left\{up^{i}q^{j}\in Z_{n}|u\in U\left(Z_{n}\right)\;and\;\left(i,j\right)\neq \left(0,0\right),\left(s,t\right)\right\},$$

where $U\left(Z_{n}\right)$ be the units set of $Z_{n}$ .

Let $x=u_{1}p^{i}q^{j},y=u_{2}p^{i^{\prime }}q^{j^{\prime }}\in Z{\left(Z_{n}\right)}^{*}$ , such that *ann*(*x*) = *ann*(*y*). Assume that (*i*, *j*) ≠ (*i*′, *j*′). Without loss of generality, we may let *i* < *i*′. There are the following cases.

**Case** **1:** *i* < *i*′ < *s*.

Since *z* = *up* ^*s*−*i*^′*q* ^*t*^ ∈ *ann*(*y*). But *xz* = *u*_1_ *up* ^*s*−*i*^′^+*i*^ *q* ^*t*+*j*^ is not divisible by *n*, a contradiction. therefore, (*i*, *j*) = (*i*′, *j*′) and [*x*] = [*y*] = *V*_*ij*_.

**Case 2:** *i* < *s* < *i*′.

Since *z* = *up* ^*s*−*i*−1^ *q* ^*t*^ ∈ *ann*(*y*). But *xz* = *u*_1_ *up* ^*s*−1^ *q* ^*t*+*j*^ is not divisible by *n*, a contradiction. therefore, (*i*, *j*) = (*i*′, *j*′) and [*x*] = [*y*] = *V*_*ij*_.

**Case 3:** *s* < *i* < *i*′.

In this case, we have *j* < *t* and *j*′ < *t*. If *j* ≠ *j*′, then *z* = *uq* ^*min*{*t*−*j*,*t*−*j*^′^}^ ∈ *ann*(*x*) or *z* = *uq* ^*min*{*t*−*j*,*t*−*j*^′^}^ ∈ *ann*(*y*) but not both. A contradiction. therefore, *j* = *j*′ and [*x*] = [*y*] = *V*_*sj*_.

Then the result is holds.

(2) Let $d_{G}^{k}\left(V_{ij}\right)$ denote the sum of distances of the vertex *V*_*ij*_ from the vertices of *G* with a distance of *k*, where *k* = 1, 2 or 3 by Proposition 1. Then

$$d_{G}\left(V_{ij}\right)=d_{G}^{1}\left(V_{ij}\right)+d_{G}^{2}\left(V_{ij}\right)+d_{G}^{3}\left(V_{ij}\right).$$

There are the following cases.

**Case 1:** *i* = 0 and 0 < *j* < *t*.

By Lemma 1 there are *V*_*ij*_ ↔ *V*_*i*′*j*′_ if and only if *i* + *i*′ ≥ *s* and *j* + *j*′ ≥ *t*. So in this case $d_{G}^{1}\left(V_{ij}\right)=j$ because *i*′ = *s* and *j*′ = *t* − 1, …, *t* − *j*. By the proof of Proposition 1, *d* (*V*_*ij*_, *V*_*i*′*j*′_) = 3 if and only if *i*′ = 1, 2, …, *s* and *j*′ = 0. So $d_{G}^{3}\left(V_{ij}\right)=3s$ . therefore

$$\begin{array}{ll} d_{G}^{2}\left(V_{ij}\right) & =2\left(\left|V\left(G\right)|-d_{G}^{1}\left(V_{ij}\right)-\frac{1}{3}d_{G}^{3}\left(V_{ij}\right)-|\left\{V_{00},V_{ij},V_{st}\right\}\right|\right)\\ & =2\left(\left(s+1\right)\left(t+1\right)-j-s-3\right). \end{array}$$

Hence, *d*_*G*_ (*V*_*ij*_) = 2 (*s* + 1) (*t* + 1) + *s* − *j* − 6.

**Case** **2:** *i* = 0 and *j* = *t*.

As case 1, $d_{G}^{1}\left(V_{ij}\right)=t$ because *i*′ = *s* and *j*′ = *t* − 1, *t* − 2, …, 0. Since *d* (*V*_*ij*_, *V*_*i*′*j*′_) = 3 if and only if *i*′ = 1, 2, …, *s* − 1 and *j*′ = 0. Then $d_{G}^{3}\left(V_{ij}\right)=3\left(s-1\right)$ . Therefore

$$\begin{array}{ll} d_{G}^{2}\left(V_{ij}\right) & =2\left(\left|V\left(G\right)|-d_{G}^{1}\left(V_{ij}\right)-\frac{1}{3}d_{G}^{3}\left(V_{ij}\right)-|\left\{V_{00},V_{ij},V_{st}\right\}\right|\right)\\ & =2\left(\left(s+1\right)\left(t+1\right)-t-\left(s-1\right)-3\right). \end{array}$$

Hence, *d*_*G*_ (*V*_*ij*_) = 2 (*s* + 1) (*t* + 1) + *s* − *t* − 7.

**Case 3:** 0 < *i* < *s* and *j* = 0.

A similar argument as in Case 1 shows that, *d*_*G*_ (*V*_*ij*_) = 2 (*s* + 1) (*t* + 1) + *t* − *i* − 6.

**Case** **4:** *i* = *s* and *j* = 0.

A similar argument as in Case 2 shows that, *d*_*G*_ (*V*_*ij*_) = 2 (*s* + 1) (*t* + 1) + *t* − *s* − 7.

**Case** **5:** $0<i\leq \left\lceil \frac{s}{2}\right\rceil -1$ and *j* ≠ 0, or $0<j\leq \left\lceil \frac{t}{2}\right\rceil -1$ and *i* ≠ 0.

Since *d* (*V*_*ij*_, *V*_*i*′*j*′_) = 1 if and only if *i*′ = *s*, *s* − 1, …, *s* − *i* and *j*′ = *t*, *t* − 1, …, *t* − *j* except *V*_*st*_. So $d_{G}^{1}\left(V_{ij}\right)=\left(i+1\right)\left(j+1\right)-1$ . In this case, $d_{G}^{3}\left(V_{ij}\right)=0$ . Therefore

$$\begin{array}{ll} d_{G}^{2}\left(V_{ij}\right) & =2\left(\left|V\left(G\right)|-d_{G}^{1}\left(V_{ij}\right)-\frac{1}{3}d_{G}^{3}\left(V_{ij}\right)-|\left\{V_{00},V_{ij},V_{st}\right\}\right|\right)\\ & =2\left(\left(s+1\right)\left(t+1\right)-\left(\left(i+1\right)\left(j+1\right)-1\right)-3\right) \end{array}$$

Hence, *d*_*G*_ (*V*_*ij*_) = 2 (*s* + 1) (*t* + 1) − (*i* + 1) (*j* + 1) − 5.

**Case 6:** $i\geq \left\lceil \frac{s}{2}\right\rceil$ and $j\geq \left\lceil \frac{t}{2}\right\rceil$ .

Since *d* (*V*_*ij*_, *V*_*i*′*j*′_) = 1 if and only if *i*′ = *s*, *s* − 1, …, *s* − *i* and *j*′ = *t*, *t* − 1, …, *t* − *j* except *V*_*st*_, *V*_*ij*_. So $d_{G}^{1}\left(V_{ij}\right)=\left(i+1\right)\left(j+1\right)-2$ . In this case, $d_{G}^{3}\left(V_{ij}\right)=0$ . Therefore

$$\begin{array}{ll} d_{G}^{2}\left(V_{ij}\right) & =2\left(\left|V\left(G\right)|-d_{G}^{1}\left(V_{ij}\right)-\frac{1}{3}d_{G}^{3}\left(V_{ij}\right)-|\left\{V_{00},V_{ij},V_{st}\right\}\right|\right)\\ & =2\left(\left(s+1\right)\left(t+1\right)-\left(\left(i+1\right)\left(j+1\right)-2\right)-3\right) \end{array}$$

Hence, *d*_*G*_ (*V*_*ij*_) = 2 (*s* + 1) (*t* + 1) − (*i* + 1) (*j* + 1) − 4.

This completes the proof of the lemma.

Remark 1 *From the above lemma, it can be easily seen that the cardinalities of the vertex set of* *G* *,* that is, |*V*(*G*)| = (*s* + 1) (*t* + 1) − 2*. So* $|V\left(Z_{2^{2}\times 3^{3}}\right)|=10$ *as shown in Example 1.*

The following theorem gives the Wiener index of $\Gamma_{E}\left(Z_{p^{s}q^{t}}\right)$ .

Theorem 2 *For distinct prime numbers* *p*, *q* *, and some* s,t∈N *. The Wiener index of the compressed zero-divisor graph* $\Gamma \left(Z_{p^{s}q^{t}}\right)$ *is*

$$\begin{array}{cc} W\left(\Gamma_{E}\left(Z_{p^{s}q^{t}}\right)\right) & =\frac{1}{2}\left(2\left(s+1\right)\left(t+1\right)\left(s+t+st\right)-\frac{1}{2}s\left(s+1\right)-\frac{1}{2}t\left(t+1\right)-\frac{s\left(s+3\right)t\left(t+3\right)}{4}\right.\\ & \;\left.-4st+\left(s-\left\lceil \frac{s}{2}\right\rceil +1\right)\left(t-\left\lceil \frac{t}{2}\right\rceil +1\right)-7\left(s+t\right)+1\right). \end{array}$$

Proof. Let *n* = *p* ^*s*^ *q* ^*t*^, and $G=\Gamma_{E}\left(Z_{n}\right)$ . we have *V*_01_, *V*_10_, …, *V*_*s*−1,*t*_, *V*_*s*,*t*−1_ are all the vertices of *G* by Lemma 2, where *V*_*ij*_ defined by (1). Then

$$\begin{array}{ll} W\left(G\right) & =\frac{1}{2}\left(\overset{t}{\underset{j=1}{\sum }}d_{G}\left(V_{0j}\right)+\overset{s}{\underset{i=1}{\sum }}d_{G}\left(V_{i0}\right)+\overset{s}{\underset{i=1}{\sum }}\overset{t}{\underset{j=1}{\sum }}d_{G}\left(V_{ij}\right)-d_{G}\left(V_{st}\right)\right)\\ & =\frac{1}{2}\left(\overset{t}{\underset{j=1}{\sum }}\left[2\left(s+1\right)\left(t+1\right)+s-j-6\right]+\overset{s}{\underset{i=1}{\sum }}\left[2\left(s+1\right)\left(t+1\right)+t-i-6\right]-2\right.\\ & \left.\;+\overset{s}{\underset{i=1}{\sum }}\overset{t}{\underset{j=1}{\sum }}\left[2\left(s+1\right)\left(t+1\right)-\left(i+1\right)\left(j+1\right)-5\right]+\overset{s}{\underset{i=\left\lceil \frac{s}{2}\right\rceil }{\sum }}\overset{t}{\underset{j=\left\lceil \frac{t}{2}\right\rceil }{\sum }}1-\left[\left(i+1\right)\left(j+1\right)-4\right]\right)\\ & =\frac{1}{2}\left(2\left(s+1\right)\left(t+1\right)\left(s+t+st\right)-\frac{1}{2}s\left(s+1\right)-\frac{1}{2}t\left(t+1\right)-\frac{s\left(s+3\right)t\left(t+3\right)}{4}-4st\right.\\ & \left.\;+\left(s-\left\lceil \frac{s}{2}\right\rceil +1\right)\left(t-\left\lceil \frac{t}{2}\right\rceil +1\right)-7\left(s+t\right)+1\right). \end{array}$$

Example 3 *Consider the ring* $R=Z_{2^{2}\times 3^{3}}$ *. The Wiener index of the compressed zero-divisor graph* $\Gamma_{E}\left(Z_{2^{2}\times 3^{3}}\right)$ *is*

$$W\left(\Gamma_{E}\left(Z_{2^{2}\times 3^{3}}\right)\right)=78$$

*By* Theorem 2*.*

## Conclusion

In this paper, we have described the structure of the graph $\Gamma \left(Z_{p^{s}\times q^{t}}\right)$ for all distinct primes *p*, *q* and s,t∈N by partition of the vertex set. Consider the partition of the vertex set into the subsets *V*_01_, *V*_10_, …, *V*_*ij*_, ⋯ , *V*_*s*−1,*t*_, *V*_*s*,*t*−1_ as seen (1). Then *V*_*ij*_ ↔ *V*_*i*′*j*′_ if and only if *i* + *i*′ ≥ *s* and *j* + *j*′ ≥ *t*. Based on this structure, we proved that the distance of two vertices of $\Gamma \left(Z_{p^{s}\times q^{t}}\right)$ are contained in the set {1, 2, 3}, and derived an explicit formula for Wiener index of the graph in Theorem 1 using the basic counting principles.

In addition, we run the formula obtained through MATLAB software and get the data in Table 1. Then, we studied the structure of the compressed zero-factor graph of $Z_{p^{s}q^{t}}$ by treating the set *V*_*ij*_ as a single vertex of the compressed zero-divisor graph $\Gamma_{E}\left(Z_{p^{s}q^{t}}\right)$ . We showed that the degree of vertex *V*_*ij*_ generally includes six cases, with the number of the vertices of the graph be (*s* + 1) (*t* + 1) − 2. Finally we derive the corresponding formula for Wiener index $W\left(\Gamma_{E}\left(Z_{p^{s}q^{t}}\right)\right)$ in Theorem 2. Of course, we can also implement it in software if needed.

**TABLE 1:** The Wiener index of $\Gamma \;\left(Z_{n}\right)$ for *n* = 2^*s*^3^*t*^.

$Z_{n}$	2 × 3	2^2^ × 3	2^3^ × 3	2 × 3^2^	2^2^ × 3^2^	2^3^ × 3^2^	2 × 3^3^	2^2^ × 3^3^	2^3^ × 3^3^
$W\left(\Gamma \left(Z_{n}\right)\right)$	4	38	210	109	504	2294	1267	5152	22136

## Data Availability

The original contributions presented in the study are included in the article/supplementary material, further inquiries can be directed to the corresponding author.
